# Antiapoptotic effect of haem oxygenase-1 induced by nitric oxide in experimental solid tumour

**DOI:** 10.1038/sj.bjc.6600830

**Published:** 2003-03-18

**Authors:** S Tanaka, T Akaike, J Fang, T Beppu, M Ogawa, F Tamura, Y Miyamoto, H Maeda

**Affiliations:** 1 Department of Microbiology, Kumamoto University School of Medicine, 2-2-1 Honjo, Kumamoto 860-0811, Japan; 2 Department of Surgery, Kumamoto University School of Medicine, 2-2-1 Honjo, Kumamoto 860-0811, Japan

**Keywords:** nitric oxide, haem oxygenase, antiapoptosis, oxidative stress, tumour growth

## Abstract

Induction of haem oxygenase-1 (HO-1) may provide an important protective effect for cells against oxidative stress. Here, we investigated the mechanism of cytoprotection of HO-1 in solid tumour with a focus on the antiapoptotic activity of HO-1. Treatment of rat hepatoma AH136B cells with the HO inhibitor zinc protoporphyrin IX (ZnPP IX) or tin protoporphyrin IX resulted in extensive apoptotic changes of tumour cells both *in vivo* and *in vitro*. Caspase-3 activity of the ZnPP IX-treated hepatoma cells increased significantly. Moreover, ZnPP IX-induced apoptosis was completely inhibited by simultaneous incubation with a specific caspase-3 inhibitor and was partially abrogated by bilirubin, a reaction product of HO. *In vivo* ZnPP IX treatment did not affect nitric oxide (NO) production and tumour blood flow. Western blot analyses showed that HO-1 expression in AH136B cells was strongly upregulated by NO donors, for example, *S*-nitroso-*N*-acetyl penicillamine and propylamine NONOate *in vitro*; conversely, it was remarkably reduced *in vivo* by pharmacological blockade of NOS. We conclude that HO-1 may function in antiapoptotic defense of the tumour, and thus it may have important protective and beneficial effects for tumour cells against oxidative stress induced by NO, which is produced in excess during solid tumour growth *in vivo*.

Haem oxygenase (HO) is the rate-limiting enzyme in haem degradation; this reaction produces biliverdin, which is subsequently converted to bilirubin by biliverdin reductase in biological systems (Tenhunen *et al*, 1968). Haem oxygenase -2 (HO-2) is the constitutive isoform of HO and is highly expressed in testis and brain under physiological conditions (Manies, 1997). Haem oxygenase -1 (HO-1), the inducible isoform of HO, also known as heat shock protein 32 (HSP32), is found at low levels in most mammalian tissues, but it is constitutively expressed in liver and spleen and is upregulated by its substrate haem (Tenhunen *et al*, 1970) and various stress-inducing stimuli such as UV light (Keyse and Tyrrell, 1989), heavy metals (Mitani *et al*, 1993), heat shock (Shibahara *et al*, 1987), hypoxia (Motterlini *et al*, 2000), and nitric oxide (NO) (Kim *et al*, 1995; Foresti and Motterlini, 1999; Bouton and Demple, 2000). Induction of HO-1 is suggested to have a cytoprotective effect against oxidative injury (Dennery *et al*, 1997; Suttner *et al*, 1999), because some bile pigments formed via HO have been shown to behave as antioxidants (Minetti *et al*, 1998; Dore *et al*, 1999).

We previously showed in an experimental solid tumour that NO produced in excess in the local area surrounding tumour cells seemed to sustain rapid tumour growth (Doi *et al*, 1996). It has been reported, however, that a particularly high output of NO from inducible nitric oxide synthase (iNOS) is potentially cytotoxic for various tumour cells (Bastian *et al*, 1994). In this context, our earlier study indicated that HO-1 is upregulated by NO produced by iNOS in an experimental solid tumour (Doi *et al*, 1999). Haem oxygenase-1 may provide important protection for tumour cells, so that tumour cell growth is sustained by HO-1 even under oxidative stress induced by excessive NO. In fact, we found that tumour growth was remarkably suppressed by the administration of zinc protoporphyrin IX (ZnPP IX, an HO-1 inhibitor) to tumour-bearing rats (Doi *et al*, 1999). However, these cytoprotective mechanisms of HO-1 remain to be identified.

It is intriguing that overexpression of HO-1 protects endothelial cells from apoptosis induced by the host's rejection reaction in a rodent cardiac transplantation model (Hancock *et al*, 1998; Soares *et al*, 1998). In the present study, we investigated the mechanism of HO-1 cytoprotection of tumour cells, with a focus on the antiapoptotic potential of HO-1, which may contribute in a critical way to tumour growth *in vivo*.

## MATERIALS AND METHODS

### Animals and implantation of AH136B tumour

AH136B tumour (rat hepatoma) cells were implanted subcutaneously (s.c.) in a dorsal site on the foot of male Donryu rats weighing 160–180 g (SLC, Inc., Shizuoka, Japan) with an inoculum size of 1 × 10^7^ cells per injection site as described previously (Doi *et al*, 1996). Tumours were allowed to grow for 14 days and usually reached a diameter of 10–15 mm. We used the tumours with about 10 mm in diameter for the present studies. The solid tumour did not cause apparent physical effects for the tumour-bearing animals: no motor dysfunctions of the limb including paralytic and shuffling gaits and no apparent symptoms of peripheral circulatory failure in the distal area of the tumour implantation site (nor necrosis) were observed during this experiment. All animal experiments were carried out with approval by the Ethical Committee at the Center for Animal Resources and Development, Kumamoto University, which met the standard required by the United Kingdom Co-ordinating Committee on Cancer Research (UKCCCR) guidelines (Workman *et al*, 1998).

### Treatment of AH136B solid tumour with ZnPP IX *in vivo*

For the treatment of AH136B tumour *in vivo*, 500 *μ*g kg^−1^ ZnPP IX (Sigma-Aldrich Fine Chemicals, St Louis, MO, USA) or copper protoporphyrin IX (CuPP IX, Frontier Scientific Inc., Logan, UT, USA) (500 *μ*g of each in 1 ml of 10% dimethyl sulphoxide (DMSO) in 0.01 M NaOH) was injected intra-arterially (i.a.) into solid tumour via the common iliac artery that is mainly feeding the tumour, so that effective delivery of these compounds is achieved. Solid tumours were obtained 24 h after injection and were stored at −80°C until use for terminal deoxynucleotidyl transferase (TdT)-mediated dUTP-biotin nick end-labelling (TUNEL) staining and for analyses of HO-1 and HSP70.

### Analysis for apoptosis and viability of AH136B cells treated with HO inhibitors *in vitro*

AH136B tumour cells, at 5 × 10^5^ cells per well of a six-well polystyrene plate (Falcon, Becton Dickinson Labware, Lincoln Park, NJ, USA), were incubated with various concentrations of the HO inhibitors ZnPP IX and tin protoporphyrin IX (SnPP IX, Frontier Scientific) (Drummond and Kappas, 1981) in Dulbecco's minimum essential medium (Invitrogen Corp., Carlsbad, CA, USA) supplemented with 10% (v v^−1^) fetal bovine serum and 0.5% nonessential amino acids (Invitrogen). After 24 h of culture, the cells were subjected to the cell viability and apoptosis assays of TUNEL staining and caspase-3 activity determination. Similarly, the cells were treated with CuPP IX, which has no direct inhibitory effect on HO activity *in vivo* (Drummond and Kappas, 1981) and is a poor HO inhibitor *in vitro* (Zakhary *et al*, 1996). Also, the effects of ZnPP IX on cultured cells were examined in the presence of bilirubin (Wako Pure Chemical Co., Ltd, Osaka, Japan) or caspase-3 inhibitor (acetyl-Asp-Met-Gln-Asp-CHO; Peptide Institute, Inc., Osaka, Japan). Cell viability was determined via the trypan blue dye exclusion assay (Kimura *et al*, 1990). Analyses for TUNEL staining and caspase-3 activity were performed as described below.

### Effect of NO on expression of HO-1 and HSP in AH136B tumours

Tumour-bearing rats were administered either *N*^ω^-nitro-L-arginine methyl ester (L-NAME, Sigma-Aldrich Fine Chemicals) or *S*-methylisothiourea sulphate (SMT, Wako Pure Chemical Co., Ltd) in 0.2 ml of 0.9% NaCl solution, given intraperitoneally (i.p.) at a dose of 6 mg kg^−1^ per day for 5 days, beginning 9 days after tumour implantation. Solid tumour tissues were obtained 1 day after the last injection of NOS inhibitor and were stored at −80°C until use for HO-1 and HSP70 Western blotting. The effect of ischaemic stress on the expression of HSP70 protein in solid tumour was also examined. Briefly, on day 14 after tumour implantation, solid tumours were resected for Western blot analysis at different times after initiation of surgical occlusion of the common iliac artery, which serves the tumour-feeding artery of the tumour-implanted side. In addition, the effect of NO on the expression of HO-1 and HSP70 was examined with the use of AH136B cells in culture treated with the NO donors *S*-nitroso-*N*-acetyl penicillamine (SNAP, Dojindo Laboratories, Kumamoto, Japan) and propylamine NONOate (CH_3_N[N(O)NO]^−^(CH_2_)_3_NH_2_^+^CH_3_) (P-NONOate, Dojindo Laboratories). Western blotting was used to analyse the levels of HO-1 and HSP70 proteins in lysate of the AH136B cells, after a 6-h incubation with the NO donors.

### TUNEL assay

Suspensions of cultured AH136B cells, treated with various compounds as just described, were spotted onto glass microscope slides and were dried at room temperature, followed by TUNEL staining. For the analysis of solid tumour tissues, 6-*μ*m-thick frozen tissue sections were prepared with a cryostat and were air-dried overnight. Apoptotic cells were detected via the TUNEL method by using an apoptosis detection kit (TACS; Trevigen Inc., Gaithersburg, MD, USA) (Ikebe *et al*, 2000).

### Caspase-3 assay of AH136B tumour cells

Caspase-3 activity in AH136B tumour cells was measured as described previously (Enari *et al*, 1996). Briefly, lysates of AH136B cells, having had treatment with various compounds or no treatment, were incubated for 30 min at 37°C with 1 *μ*M MOCAc-Asp-Glu-Val-Asp-Ala-Pro-Lys(Dnp)-NH_2_ fluorescent substrate (Peptide Institute). Ac-DMQD-CHO, caspase-3 inhibitor (Peptide Institute), was added to the reaction mixture at a concentration of 10 *μ*M. Caspase-like amidolytic activity for the peptidyl substrate was measured fluorometrically and caspase activity was determined by subtracting the fluorescence values obtained in the presence of inhibitor.

### Measurement of HO activity in tumour cells and solid tumours

Haem oxygenase activity in AH136B tumour cells and solid tumours was quantified with use of the microsomal fraction extracted from each sample according to our previously described method (Doi *et al*, 1999). The reaction mixture for the measurement of HO activity was composed of microsomal protein (1 mg), cytosolic fraction of rat liver (1 mg protein) as a source of biliverdin reductase, 33 *μ*M hemin, and 333 *μ*M NADPH of 1 ml of 90 mM potassium phosphate buffer, pH 7.4. The bilirubin formed in the reaction (15 min at 37°C) was quantified spectroscopically (Doi *et al*, 1999).

### *In vivo* microdialysis

This technique was employed to assess the NO production in tumour tissue *in vivo* (Ohta *et al*, 1994). A microdialysis probe (straight type, outer diameter 220 *μ*m, length 5 mm, cellulose membrane, cutoff 50 000 Da; Eicom Corp., Kyoto, Japan) was implanted in the solid tumour, and was perfused using a microsyringe pump (ESP-64, Eicom Corp.). After an equilibration period (1 h), dialysate was collected in polyethylene tubes for 15 min, followed by measurement of NO_2_^−^ and NO_3_^−^ by using an NO_*x*_ analyzer (ENO-10, Eicom Corp.) (Akaike *et al*, 1997).

### Measurement of tumour blood flow

Tumour blood flow was measured before and 1, 3, 6, 12, and 24 h after injection of 500 *μ*g kg^−1^ ZnPP IX via the tumour-feeding artery or after i.p. injection of 6 mg kg^−1^ L-NAME. A laser Doppler flowmeter (Laser Flow Meter, ALF21, Advance, Tokyo, Japan) was used for measurement of the tumour blood flow via a probe needle placed into the tumour tissue as described earlier (Ikebe *et al*, 2000).

### Western blotting for expression of HO-1 and HSP70

Lysates of AH136B cells treated or untreated with various reagents were prepared as described previously (Doi *et al*, 1999). HSP70 was induced by a standard heat shock treatment at 42°C for 30 min followed by a 6-h incubation under normal culture conditions as described above. The supernatant of homogenates (10 000 **g**, 30 min) of tumour tissues treated with ZnPP IX (500 *μ*g kg^−1^, i.a.), NOS inhibitors, or vehicle was subjected to Western blot analysis for HSP70. The microsomal fraction obtained by ultracentrifugation was used for the HO-1 analysis. Total protein (25 *μ*g each) in cell lysates or tissue homogenates was used for the Western blotting with a monoclonal antibody to HSP70 (SPA-810, Stressgen, Victoria, BC, Canada), which specifically recognises the inducible but not the constitutive isoforms of the HSP70 family in mammals, or a polyclonal antibody to HO-1 (OSA-150, Stressgen). The protein band that reacted immunologically with the antibody was visualised by using the ECL system (Amersham International plc, Buck, UK).

### Statistical analysis

Data are shown as means±s.e. Statistical difference was analysed by the use of the two-tailed unpaired *t*-test and by ANOVA. A *P*-value of <0.05 was considered statistically significant.

## RESULTS

### Zinc protoporphyrin IX -induced apoptosis and inhibition of HO activity in AH136B solid tumours

**Figure 1:**
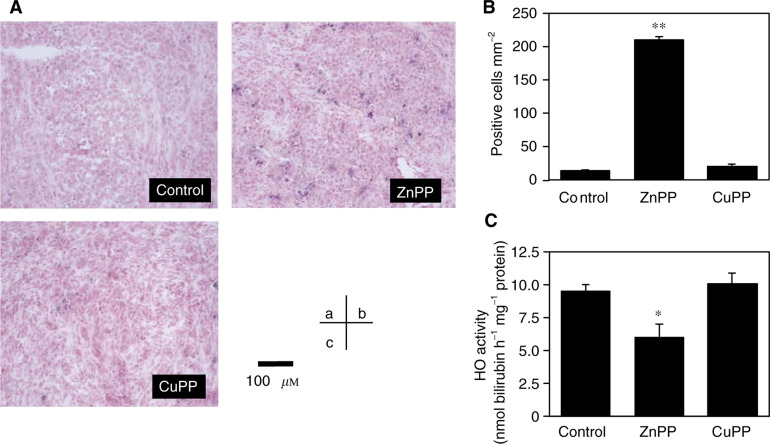
Apoptosis induction and change in HO activity after treatment with ZnPP IX of AH136B solid. Apoptosis (**A, B**) and HO activity (**C**) were assessed on day 14 after tumour implantation. Each specimen was analysed via TUNEL staining 24 h after treatment with vehicle (the control) (**A, a**), with ZnPP IX (500 *μ*g kg^−1^ i.a.) (**A, b**), or with CuPP IX (500 *μ*g kg^−1^ i.a.) (**A, c**). Quantitative analysis of TUNEL-positive cells in each specimen is shown (**B**). TUNEL-positive cells were counted in four different fields of magnification at × 100 per sample, and then the number of positive cells per mm^2^ was calculated. Haem oxygenase activity of the solid tumour was measured after treatment in the same manner as in the TUNEL analysis (**C**). ^**^P<0.05, ^**^P<0.01 *vs* control (*n*=3 for each group). Data are means±s.e. See text for details.

Strong staining of TUNEL-positive cells was detected in ZnPP IX-treated tumour tissue (Figure 1A, B

); however, the vehicle (control) (Figure 1A, A) and the CuPP IX-treated (Figure 1A, C) tumour tissues showed only negligible staining. Morphometric analysis of TUNEL-positive cells in AH136B solid tumours revealed a significantly higher number of TUNEL-positive cells in the ZnPP IX-treated group (210±6 mm^−2^) compared with the control group (14±2 mm^−2^) and the CuPP IX-treated group (20±4 mm^−2^) (Figure 1B).

A significant, although not complete, inhibition of HO activity in solid tumour tissue was obtained by ZnPP IX treatment (Figure 1C). The CuPP IX-treated group showed almost the same level of HO activity as the control group. These results suggest that ZnPP IX-induced apoptosis in AH136B solid tumour is mediated through the inhibition of HO activity by ZnPP IX.

### Zinc protoporphyrin IX-induced apoptosis and inhibition of HO activity in AH136B cells in culture

**Figure 2:**
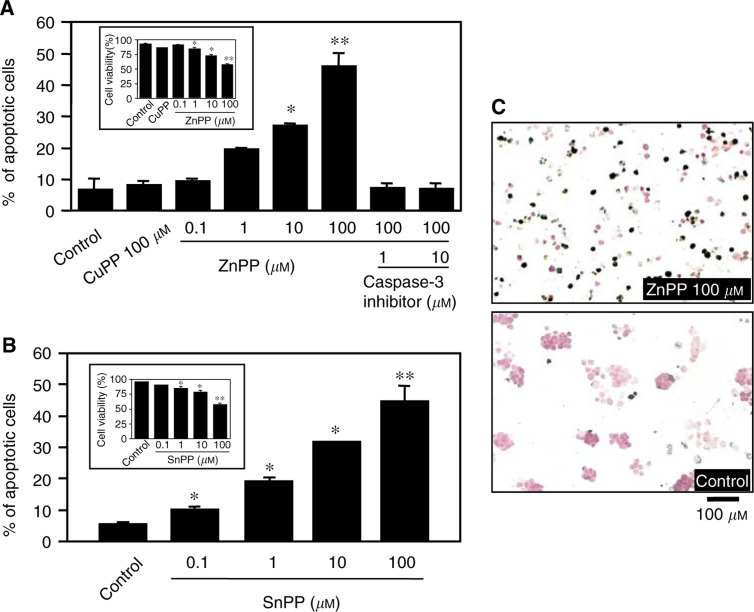
Zinc protoporphyrin IX-induced apoptosis of AH136B cells *in vitro*. AH136B cells were incubated for 24 h with indicated concentrations of ZnPP IX, with or without caspase-3 inhibitor (**A**) or SnPP IX (**B**), or with 100 *μ*M CuPP IX. (**A, B**) TUNEL-positive cells were counted in four different fields of magnification at × 100 per sample, and then the percentage of positive cells was calculated. ^**^P<0.05 *vs* control (*n*=3 for each group). Cell viability after the same treatment is shown in the inset. ^**^P< 0.05, ^**^P<0.01 *vs* control (*n*=4 for each group). Data are means±s.e. (**C**) Representative TUNEL staining of control and ZnPP IX-treated tumour cells. See text for details.

Cultured AH136B cells were incubated for 24 h with indicated concentrations of ZnPP IX (with or without a caspase-3 inhibitor) or SnPP IX, or with 100 *μ*M CuPP IX (Figure 2

). The number of TUNEL-positive cells in the CuPP IX-treated group (8.1±1.3%) was comparable to that of the control group (6.6±3.8%). In contrast, 1 *μ*M and higher concentrations of ZnPP IX potentiated the induction of apoptosis in AH136B cells in a concentration-dependent manner (46.7% at 100 *μ*M ZnPP) (Figure 2A). A similar trend for the induction of apoptosis was observed with another HO inhibitor, SnPP IX (Figure 2B). The ZnPP IX-induced apoptosis was completely inhibited by simultaneous incubation with a specific caspase-3 inhibitor (at 1 or 10 *μ*M). The viability of tumour cells was inversely decreased in a concentration-dependent manner after ZnPP IX treatment (inset in Figure 2A, B). Figure 2C shows representative TUNEL staining of control and ZnPP IX-treated tumour cells (100 *μ*M ZnPP IX), illustrating a considerable number of TUNEL-positive cells in the ZnPP IX-treated group compared with the control staining.

**Figure 3:**
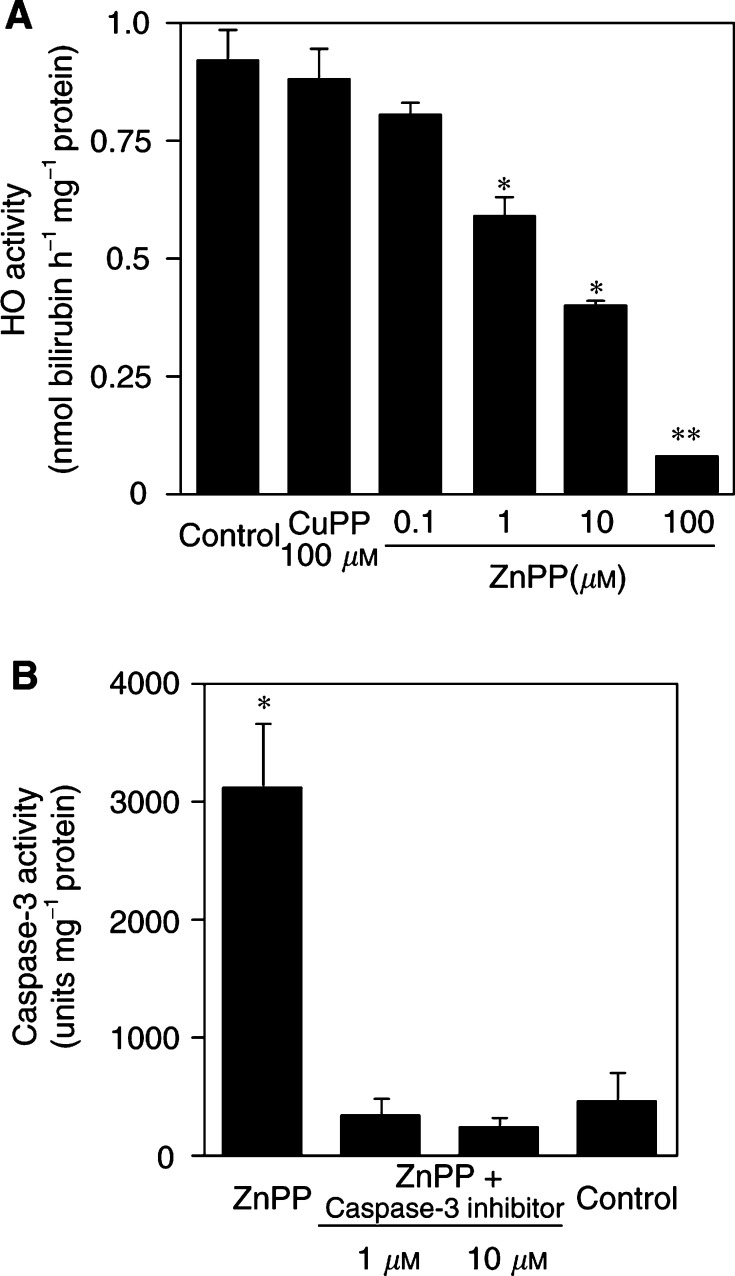
Effect of ZnPP IX on HO activity and caspase-3 activity of AH136B cells cultured *in vitro*. Haem oxygenase activity was measured 24 h after incubation with vehicle (control), ZnPP IX, or CuPP IX (**A**). Similarly, cells were treated with 100 *μ*M ZnPP with or without caspase-3 inhibitor (1 or 10 *μ*M) for 24 h (**B**). Caspase-3 activity was measured fluorome-trically in cell extracts by using a fluorescent substrate. ^**^P<0.05, ^**^P<0.01 *vs* control (*n*=3 for each group). Data are means±s.e. See text for details.

Zinc protoporphyrin IX treatment resulted in a remarkable reduction of HO activity in the cells (Figure 3A

), which correlated very well with the level of apoptosis caused by ZnPP IX treatment (Figure 2). Copper protoporphyrin IX, which showed no appreciable apoptotic effect, also did not affect cellular HO activity (Figure 3A). In addition, caspase-3 activity in AH136B cells was greatly increased after 24 h of incubation with 100 *μ*M ZnPP IX, and this increase was totally nullified by simultaneous incubation with caspase-3 inhibitor at concentrations of 1 and 10 *μ*M (Figure 3B). These results are consistent with the finding from the *in vivo* solid tumour study and indicate again that pharmacological blockade of HO activity induces apoptotic change of the AH136B tumour cells.

### Protective effect of bilirubin against ZnPP IX-induced apoptosis of AH136B cells

**Figure 4:**
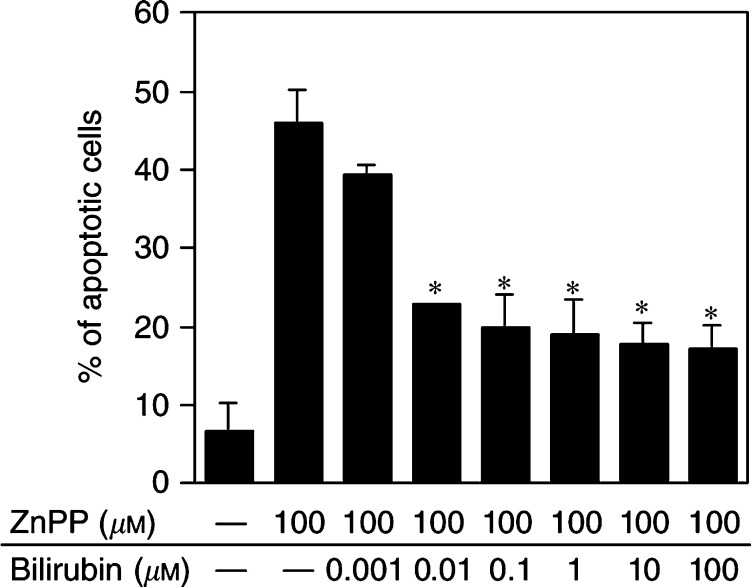
Protective effect of bilirubin against ZnPP IX-induced apoptosis of AH136B cells. AH136B cells were incubated for 24 h with 100 *μ*M ZnPP IX in the presence or absence of indicated concentrations of bilirubin. TUNEL-positive cells were counted in four different fields of magnification at × 100 per sample, and then the percentage of positive cells was calculated. ^**^P<0.05 *vs* ZnPP IX alone (*n*=3 for each group). Data are means±s.e. See text for details.

We further examined the effect of bilirubin, which is biologically derived from biliverdin, an important enzymatic reaction product of HO (Figure 4

). There was a trend toward inhibition of ZnPP IX-induced apoptosis even at 0.001 *μ*M bilirubin, although it was not statistically significant. Addition of 0.01 *μ*M or higher concentrations of bilirubin caused a marked reduction in the number of TUNEL-positive cells. This suppressive effect of bilirubin on ZnPP IX-induced apoptosis of AH136B cells was not truly dose dependent, however, and one-third of the TUNEL-positive cells remained unaffected at the highest concentration of bilirubin (100 *μ*M).

### Effect of ZnPP IX treatment on blood flow and NO production in AH136B solid tumours

**Figure 5:**
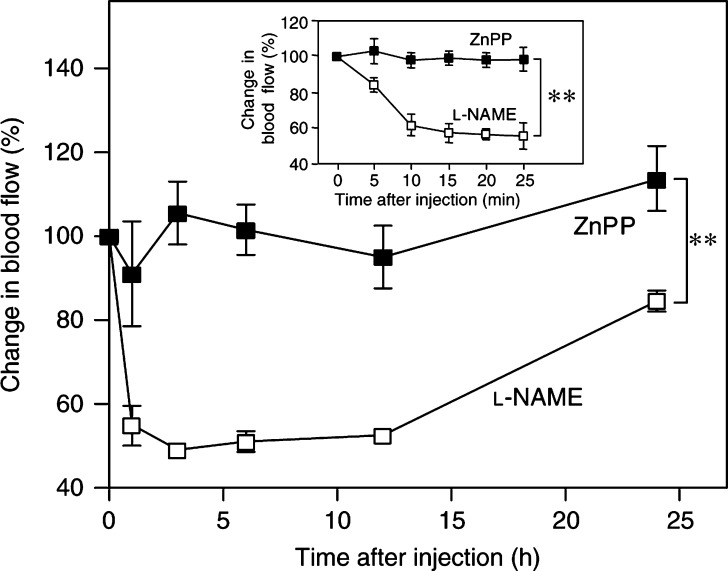
Tumour blood flow after ZnPP IX or L-NAME treatment. Tumour blood flow was measured in AH136B solid tumour on day 14 after tumour implantation. At the indicated times after injection of 500 *μ*g kg^−1^ ZnPP IX (i.a.) or 6 mg kg^−1^ L-NAME (i.p.), a laser Doppler flowmeter and a probe needle were used to measure blood flow. Relative changes in tumour blood flow after injection of ZnPP IX or L-NAME are shown. Inset, change in tumour blood flow during early period after treatment (within 25 min). ^**^P<0.01 by ANOVA between the groups (*n*=3 for each time point). Data are means±s.e. See text for details.

Carbon monoxide (CO) generated by HO has been demonstrated to be a potential endogenous modulator of vascular tone in the liver (Suematsu *et al*, 1994). Hence, we examined whether ZnPP IX treatment modulated blood flow in this solid tumour through the inhibition of CO biosynthesis (Figure 5

). Tumour blood flow was not affected by ZnPP IX in this model, although it was significantly decreased 1, 3, 6, and 12 h after administration of L-NAME. Moreover, ZnPP IX did not appreciably affect NO production in AH136B solid tumours, as evidenced by the microdialysis technique to analyse the amount of NO_2_^−^ and NO_3_^−^ *in situ* generated in the dialysate of the tumour tissues: 3.5±1.1 and 3.2±1.4 *μ*M of NO_2_^−^ and NO_3_^−^ were generated for 15 min in 10 *μ*l dialysates of the tumour tissues before and 24 h after ZnPP IX treatment, respectively (*n*=5 for each group; *P*=0.67). No appreciable suppression for NO production was observed at any time points (1, 2, 3, 6, and 12 h) after ZnPP IX administration (data not shown).

### Expression of HSP70 and HO-1 proteins in AH136B cells and solid tumours

**Figure 6:**
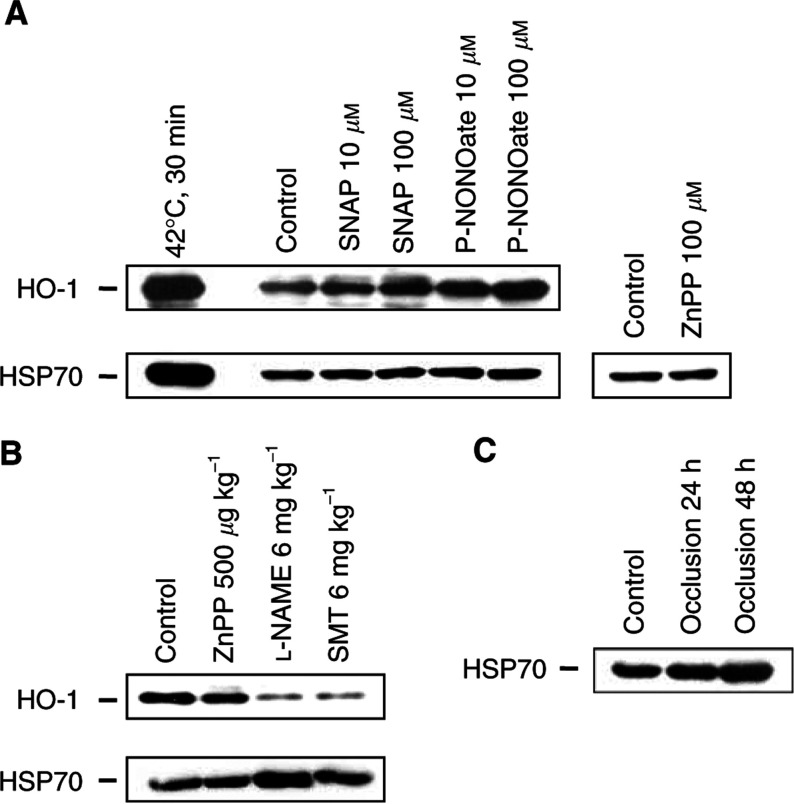
Western blot analysis of HSP70 and HO-1 proteins in AH136B cells and solid tumours. (**A**) Cells were incubated with SNAP (10 or 100 *μ*M), P-NONOate (10 or 100 *μ*M), or ZnPP IX (100 *μ*M) for 6 h or, as a positive control, were treated by heat at 42°C for 30 min, followed by incubation at 37°C for 6 h. (**B**) AH136B solid tumours were treated with 0.9% NaCl solution (control), ZnPP IX (500 *μ*g kg^−1^ i.a.), L-NAME (6 mg kg^−1^ day^−1^ for 5 days i.p.), or SMT (6 mg kg^−1^ day^−1^ for 5 days i.p.). (**C**) Solid tumours were resected at 24 or 48 h after initiation of surgical occlusion of the common iliac artery, which serves the tumour-feeding artery of the tumour-implanted side. Control rats were treated by sham operation. Then, expression of HSP70 and HO-1 proteins was detected by Western blotting by using a monoclonal antibody to mammalian inducible HSP70 or a polyclonal antibody to rat HO-1. Each data point shown represents three independent experiments. See text for details.

Western blotting was performed to analyse the expression of HO-1 protein and another important HSP, HSP70, in AH136B tumour cells and solid tumour tissues (Figure 6

). Not only AH136B cells in culture but also AH136B solid tumours grown *in vivo* show constitutive expression of HSP70 and HO-1 proteins without any particular stimulation. The levels of expression of both of these proteins were strongly upregulated by heat shock treatment. The expression of HO-1 was also greatly enhanced by NO generated exogenously from SNAP or P-NONOate added to the culture of the AH136B tumour cells (Figure 6A, upper panel), whereas the same treatment produced no measurable change in HSP70 expression (Figure 6A, lower panel). Also, ZnPP IX treatment of the cultured tumour cells had no effect on HSP70 expression (Figure 6A, lower panel). Similarly, ZnPP IX administration to solid tumour tissues did not influence HO-1 or HSP70 expression *in vivo* (Figure 6B), suggesting no compensatory regulation of HO-1 and HSP70.

Haem oxygenase-1 expression in AH136B tumour tissues *in vivo* was strongly attenuated by treatment with L-NAME or SMT (Figure 6B, upper panel), which is consistent with the *in vitro* result of strong upregulation of HO-1 in AH136B tumour cells induced by NO. In contrast, the level of HSP70 expression was increased by the same NOS inhibitor treatment (Figure 6B, lower panel). This upregulation of HSP70 by NOS inhibitors was possibly caused by hypoxic stress of the solid tumour tissues produced by blockade of NO biosynthesis. The same HSP70 upregulation occurred after an ischaemic insult caused by occlusion of the tumour-feeding artery (Figure 6C). These data indicate that HO-1 expression was regulated mainly by NO generated endogenously in the solid tumour tissue, whereas HSP70 expression was modulated through a separate mechanism, possibly dependent on a hypoxic cellular signalling pathway of the tumour cells.

## DISCUSSION

In the present study, we clearly demonstrate that HO-1 induced by NO had a potent antiapoptotic function in an experimental AH136B solid tumour in rats. Earlier studies suggested a cytoprotective effect of HO-1, that is, inhibition of apoptosis in transplant injury during organ rejection and of TNF-*α*-induced apoptosis in fibroblasts (Hancock *et al*, 1998; Soares *et al*, 1998; Petrache *et al*, 2000). Here, we found that ZnPP IX induced the apoptosis of AH136B cells both *in vivo* and *in vitro* through inhibition of HO-1 activity, and HO-1 activity was upregulated by NO generated in the tumour tissues. It was recently reported that ZnPP IX had a direct cytotoxic effect through apoptosis induction, regardless of HO inhibition (Lutton *et al*, 1997). Another potent HO inhibitor is, however, shown not to have such a direct cytotoxic (apoptotic) effect (Yang *et al*, 2001). In our study, not only ZnPP IX but also SnPP IX, which inhibits HO specifically without direct cytotoxic action, showed potent apoptosis-inducing potential in AH136B tumour cells. In addition, ZnPP IX treatment of the rats did not affect the blood flow in the tumour tissue (Figure 5), suggesting that ZnPP IX does not have a significant inhibitory effect at least on the soluble guanylate cyclase activity as seen in blood flow (via NO-cGMP pathway), which is a major inducer for vasorelaxation. It is thus reasonable to conclude that the HO-1 expressed by the tumour cells may have a cytoprotective function, so that the solid tumour can grow effectively during the oxidative stress that occurs as the tumour grows.

To further confirm the antiapoptotic activity of HO-1, we performed a separate experiment by using a small interfering RNA (siRNA) to suppress specifically the expression of HO-1. Specifically, endogenous HO-1 mRNA was targeted in cultured SW480 cells, a cell line of human colon adenocarcinoma, by transfecting a 21-nucleotide duplex siRNA derived from the HO-1 nucleotide sequence according to the method reported recently (Elbashir *et al*, 2001). Small interfering RNA-treated SW 480 cells underwent a significant apoptotic change, which was almost comparable to the effect of ZnPP IX added to the cell culture (data not shown). These data indicate a clear cause and effect relation for the HO-1-mediated antiapoptotic effect.

It is of considerable importance that bilirubin, which is converted from biliverdin, a product of HO, significantly decreased the number of TUNEL-positive AH136B cells treated with ZnPP IX. Bilirubin appears to be one of the most abundant endogenous antioxidants in mammalian tissues and accounts for most of the antioxidant activity in human serum (Minetti *et al*, 1998). Bilirubin displayed potent scavenging activity against various oxidants including superoxide, peroxyl radical, and peroxynitrite (Minetti *et al*, 1998; Dore *et al*, 1999). More importantly, a neuroprotective effect of bilirubin derived from HO-2 has been shown for hydrogen peroxide-induced cytotoxicity in cultured rat primary neuronal cells (Dore *et al*, 1999). Since oxidative stress is suggested to be a potential mediator of apoptosis induction (Buttke and Sandstrom, 1994), it is reasonable that the antioxidant activity of bilirubin just mentioned may antagonise the apoptosis elicited by HO inhibitor treatment in AH136B tumours.

However, bilirubin is also reputed to be a potentially toxic agent, particularly when it accumulates in the serum of neonates and causes jaundice. At high concentrations, bilirubin is deposited in selected brain regions and produces neurotoxicity associated with kernicterus (Gourley, 1997). In our present study, no clearly dose-dependent inhibitory effect of bilirubin on ZnPP IX-induced apoptosis of AH136B cells was observed with bilirubin concentrations at more than 0.1 *μ*M. This incomplete inhibition of apoptosis by bilirubin may be attributed to the cytotoxic effect of bilirubin itself rather than its antioxidant activity.

Another explanation for the saturated antiapoptotic effect of bilirubin is that CO, which is a catalytic by-product of HO-1 activity, may be involved in the cytoprotective mechanism of HO-1. In fact, Choi's group demonstrated that TNF-*α*-induced apoptosis in cultured fibroblasts was inhibited by CO exposure (Petrache *et al*, 2000). Moreover, a low concentration of CO can provide protection against hyperoxic lung injury *in vivo* (Otterbein *et al*, 1999).

CO has been suggested to function as a neurotransmitter and also as an endogenous modulator of vascular perfusion in the liver through a mechanism analogous to that of NO (Suematsu *et al*, 1994). Zinc protoporphyrin IX was previously shown to reduce the tumour blood flow relatively selectively via mechanisms unrelated to HO inhibition (Tozer *et al*, 1998). It is important to confirm the lack of NOS inhibition by ZnPP IX, because NO has a biological effect very similar to HO-1 in terms of sustaining the solid tumour growth (Doi *et al*, 1996) as well as antioxidant effect. However, ZnPP IX affected neither tumour blood flow nor NO biosynthesis in the AH136B solid tumour tissue; whereas, tumour blood flow was significantly decreased by treatment with an NOS inhibitor (L-NAME). These data suggest that NO rather than CO derived from HO may be the dominant modulator of tumour blood flow, at least in this solid tumour model.

Although several reports have shown a cytoprotective effect of HO-1 (Dennery *et al*, 1997; Suttner *et al*, 1999), contradictory effects of the biological consequences of overexpression of HO-1 have also been reported. Specifically, a study by Suttner and Dennery (1999) indicated that overexpression of HO-1 could exacerbate the oxidative stress of cells, and they revealed a critical role of reactive iron (ferric iron, Fe^2+^) released during HO-catalytic decomposition of the porphyrin ring of haem in determining the consequence of HO expression.

In our own study, we assumed that some other cytoprotective system may occur to compensate for such a disadvantage of HO-1 induction in our experimental solid tumour. HSP70, which is one of the most important HSPs ubiquitously distributed in mammalian systems (Schlesinger, 1990; Xu *et al*, 1997), was expressed by AH136B cells and solid tumours. However, although HSP70 has been reported to be induced by NO exposure (Xu *et al*, 1997), our present analysis of HSP70 with NO donors and NOS inhibitors *in vitro* and *in vivo* showed that NO does not participate in HSP70 upregulation in AH136B cells. Thus, we suggest that HSP70 expression in AH136B tumours may be positively regulated by ischaemia or hypoxia through a mechanism different from HO-1 induction involving NO.

AH136B experimental solid tumour tissues produce a high amount of NO, which seems to sustain rapid tumour growth, as we reported previously (Doi *et al*, 1996). NO mediates angiogenesis and enhanced vascular permeability in solid tumour (Jenkins *et al*, 1995; Wu *et al*, 1998), and is implicated in the maintenance of blood flow in the neovasculature of the tumour (Tozer *et al*, 1998). In addition, it has been reported that NO inhibits apoptosis and its mechanism appears to be via inhibition of the caspase protease cascade (Mannick *et al*, 1994; Ogura *et al*, 1998). However, no significant modification of NO production was observed with ZnPP IX treatment, as determined by microdialysis-based NO_2_^−^ and NO_3_^−^ measurement in our experimental model, indicating that the apoptotic change in the AH136B solid tumours after ZnPP IX treatment depended mostly on the specific suppression of HO activity.

In conclusion, our current study indicates that HO-1 may function as an antiapoptotic defense system for the tumour, and it may also have important protective and beneficial effects for tumour cells against oxidative stress occurring during rapid growth of solid tumour *in vivo*. Thus, HO-1 may become a potential target for cancer chemotherapeutic agents, particularly in combinations with conventional agents. The present study warrants further investigation to develop new tactics for antitumour treatment with the use of HO inhibitors such as ZnPP IX or its polymer-conjugated derivatives with improved pharmacological properties (Sahoo *et al*, 2002).
